# Two Cases of Intestinal Tuberculosis With Severe and Fatal Gastrointestinal Bleeding: Is the Indication for Intravenous Therapy Essential?

**DOI:** 10.7759/cureus.83289

**Published:** 2025-05-01

**Authors:** Vitor C Pereira Machado, Nicolas A Weidebach, Richard Calanca, Igor G Oviedo Garcia, Cesar C Ponce, Rosely Antunes Patzina, Jose C Ardengh

**Affiliations:** 1 Infectious Diseases, Instituto de Infectologia Emilio Ribas, São Paulo, BRA; 2 Digestive Endoscopy, Instituto de Infectologia Emilio Ribas, São Paulo, BRA; 3 Pathology, Instituto de Infectologia Emilio Ribas, São Paulo, BRA; 4 Pathology, Universidade de São Paulo, São Paulo, BRA; 5 Gastrointestinal Endoscopy, Hospital das Clínicas de Ribeirão Preto, Ribeirão Preto, BRA; 6 Image Diagnosis, Universidade Federal de São Paulo, São Paulo, BRA

**Keywords:** acquired immunodeficiency syndrome, colon, gastrointestinal tuberculosis, intravenous tuberculostatic drugs, lower gastrointestinal bleeding

## Abstract

These two cases involve two patients with intestinal tuberculosis and severe lower gastrointestinal (GI) bleeding. The first case was a young woman who lived with HIV/AIDS and disseminated tuberculosis with wasting, pulmonary, and abdominal involvement that, despite the usual tuberculostatic treatment, evolved with a massive GI bleed. Her colonoscopy showed granulomatous colitis with active bleeding, and her biopsy showed positive acid-fast bacilli, confirming the diagnosis. Despite oral tuberculosis and endoscopic hemorrhage control therapies, the patient suffered recrudescent bleeding, evolved to refractory hemorrhagic shock, and died. The other case was that of a young man with no immunological impairment who had disseminated tuberculosis with intestinal impairment. This patient developed severe lower GI bleeding after six days of oral therapy and was subsequently transitioned to full intravenous (IV) treatment. Follow-up colonoscopies were performed after two weeks and one month of IV therapy. The first examination revealed ulcers covered with fibrinous layers and no signs of recent bleeding, while the second showed almost complete healing of the lesions. The patient demonstrated clinical improvement with no recurrence of bleeding and was discharged for outpatient follow-up after a total of three months of hospitalization. These two clinical cases highlight the importance of a precocious diagnosis, as well as the right interventions in patients with disseminated tuberculosis with GI involvement, emphasizing the importance of intravenous therapy over oral therapy.

## Introduction

Patients with HIV/AIDS are at increased risk of developing extrapulmonary and disseminated forms of tuberculosis (TB), including intestinal involvement. This susceptibility is primarily attributed to the dysfunction of Mycobacterium tuberculosis-specific CD4+ and CD8+ T cells caused by HIV infection, which impairs the production of essential cytokines, such as IL-2, IFN-γ, and TNF-α [1]. Patients with this form of TB usually have nonspecific signs and symptoms, such as diffuse abdominal pain, diarrhea, weight loss, low-grade fever, and abdominal distention [2-4]. Intestinal complications present a challenge to clinicians due to a need for strong clinical suspicion in this population [2,4,5]. Peritoneal and intestinal TB complications include adhesions, perforations, obstructions, abscesses, peritonitis, and, more rarely, hemorrhages [6,7]. In some cases, when intestinal perforation or severe obstruction is present, surgical treatment is required. Surgery may also be indicated when the disease is refractory to oral treatment or the patient is in danger of imminent death [8-11]. Despite the rarity of these complications, reported cases are significant because of their potentially severe consequences, including circulatory shock and death [10,12,13]. In cases of severe gastrointestinal (GI) involvement, particularly when bleeding is present, intravenous (IV) treatment becomes crucial. Considering both the impaired intestinal absorption of oral therapies and the prevention of recurrent bleeding, IV therapy, either alone or in combination with oral treatment, has demonstrated favorable outcomes [10,11]. No study or official guideline establishes an exact duration for IV therapy. Instead, the decision regarding its length should be individualized, taking into account the severity of the disease, the patient's clinical response, and the feasibility of a safe transition to oral therapy.

The authors of this report, considering the two cases described with different outcomes and following a multidisciplinary discussion, suggest that IV treatment may have been a key factor that shifted the outcome in the second case from fatality to clinical improvement. These cases highlight the importance of recognizing this complication in diverse populations and stress the need for protocols ensuring early diagnosis, appropriate treatment, and follow-up to reduce morbidity and mortality.

## Case presentation

Case 1

A 19-year-old needlewoman presented herself to the infectious disease emergency department with a six-month history of an approximately 15 kg weight loss and a two-month history of productive yellow sputum cough. Two weeks prior to admission, she had developed diffuse abdominal pain and a watery diarrhea with one episode of a small quantity of blood in her stool. About 14 years ago, the patient was diagnosed with HIV, which she said was treated regularly with tenofovir, lamivudine, and dolutegravir, although there were no control comprobatory viral load exams conducted at that time. She was torporous, dehydrated, pale, and afebrile, but with a heart rate of 143 beats per minute. An oral cavity search revealed white plaque through the mucosa, and lung sounds revealed diffuse rhonchi and focal rales. Her abdomen was flat but tender and painful at examination, without signs of clinical peritonitis. Both the liver and spleen were painful at 2 cm from the costal margins. Extremities had a discrete bilateral compressible edema.

**Figure 1:**
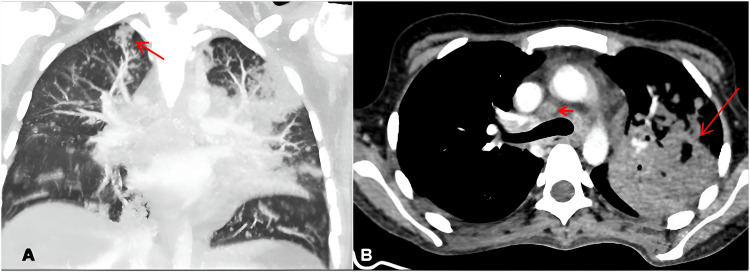
Patient's chest CT scan - Case 1 Chest CT images exhibiting diffuse centrilobular micronodules in the coronal view (Figure 1A), showing a tree-in-bud (TIB) pattern in the upper right lobe (red arrow); axial image (Figure 1B) showing mediastinal and hilar lymphadenopathy, measuring up to 18 mm in its smaller axis (arrowhead), as well as consolidation surrounded by adjacent restrictive pulmonary atelectasis in the upper left lobe (red arrow).

A computerized tomography (CT) scan showed centrilobular distribution of pulmonary nodules and micronodules, as well as an excavated consolidative lesion in her upper left lobe and mediastinal and hilar lymphadenopathies measuring up to 1.8 cm (Figure 1).

**Figure 2:**
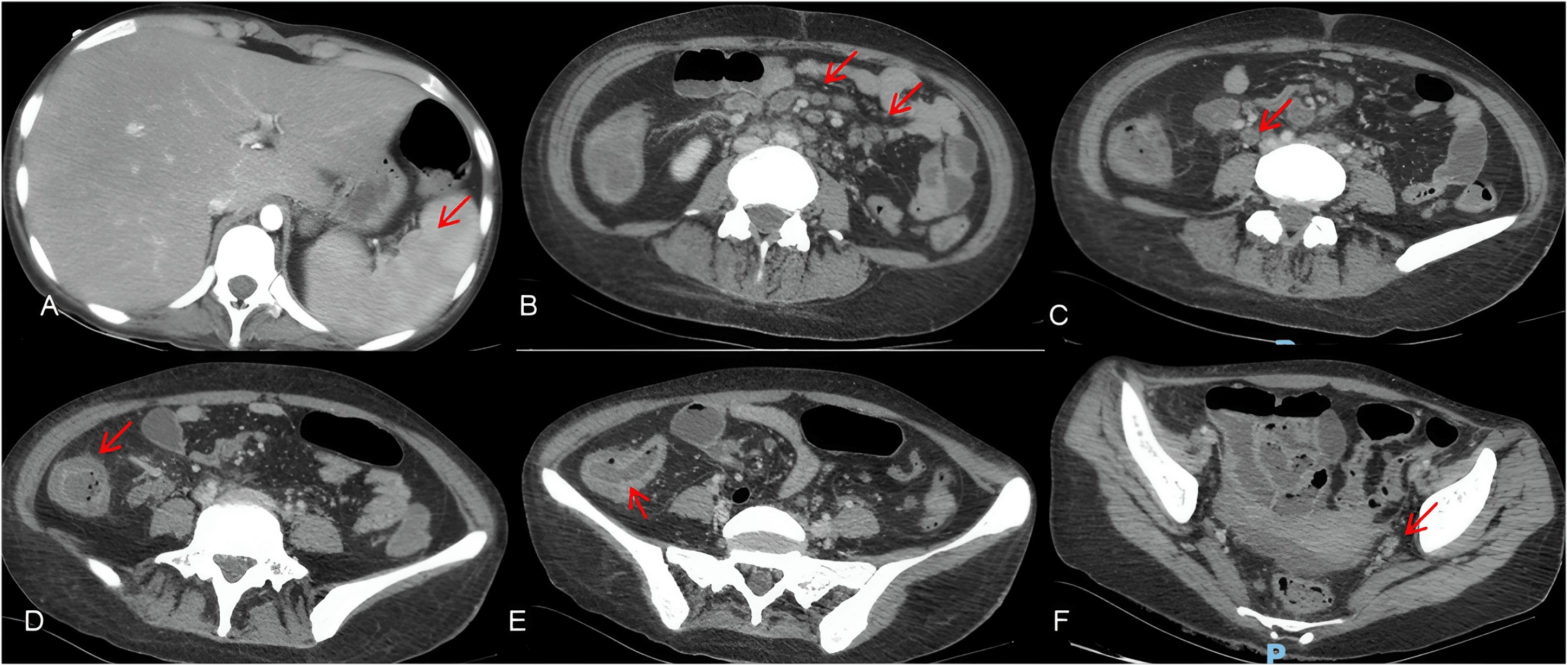
Patient's abdominal CT scan with contrast Abdominal CT scan with contrast showing liver and spleen enlargement with multiple hypoattenuating lesions on the splenic parenchyma, without contrast enhancement, suggestive of tuberculous microabscesses (red arrow in Figure 2A). Figures 2D and 2E exhibit intense cecal and ascending colon wall thickening by red arrows. Figures 2B, 2C, and 2F show multiple-sized, confluent lymphadenopathies in the hepatic hilum and the peripancreatic, periceliac, mesenteric, pericaval, iliac, and retroperitoneal chains, measuring up to 14 mm on on the smaller axis, most of them showing central hypodensity, suggestive of necrosis (thickening, red arrows).

Abdominal CT showed discrete hepatosplenomegaly with splenic abscesses and diffuse colon wall thickening, especially in its ascending portion, with contrast enhancement and multiple intra-abdominal lymphadenopathies (Figure 2).

Admission peripheral blood cultures were positive for M. tuberculosis complex, and sputum was positive for direct acid-fast bacilli staining, as well as for the rapid molecular TB test without detection of rifampin-resistant genes.

The other laboratory analysis, available in Table 1, revealed abnormalities. Hemoglobin (9.6 g/dL) and hematocrit (29.3%) were both markedly reduced, indicating anemia. Leukocyte count was elevated at 13.5 × 10³/mm³ (reference: 4.00-11.00 × 10³/mm³), suggestive of an inflammatory or infectious process. C-reactive protein (CRP) was significantly increased at 247.6 mg/L (reference: <5.0 mg/L), further supporting the presence of systemic inflammation. Lactate dehydrogenase (LDH) was also markedly elevated at 768 U/L (reference: 120-246 U/L), which may reflect tissue injury. Aspartate aminotransferase (GOT) was elevated at 73 U/L (reference: <36 U/L), whereas alanine aminotransferase (GPT) remained within normal limits at 28 U/L (reference: <35 U/L). Total bilirubin was increased at 2.2 mg/dL (reference: 0.2-1.2 mg/dL), with direct bilirubin predominance. In contrast, platelet count (308 × 10³/mm³), serum creatinine (0.7 mg/dL), and urea (19 mg/dL) were within normal reference ranges.

**Table 1:** Laboratory analysis on admission - Case 1 CRP: C-reactive protein; GPT: glutamate pyruvate transaminase; GOT: glutamic oxaloacetic transaminase; LDH: lactate dehydrogenase; WBC: white blood cells

Exam	Result	Reference Value
Hemoglobin	9.6 g/dL	13.5-17.5 g/dL
Hematocrit	29.3%	40.0%-52.0%
WBC	13.5 x 10³/mm³	4.00-11.00 x 10³/mm³
Platelets	308 x 10³/mm³	140-450 x 10³/mm³
CRP	247.6 mg/L	< 5.0 mg/L
LDH	768 U/L	120-246 U/L
Creatinine	0.7 mg/dL	0.52-1.04 mg/dL
Urea	19 mg/dL	15-36 mg/dL
GOT	73 U/L	< 36 U/L
GPT	28 U/L	< 35 U/L
Bilirubin	TBIL 2.2 mg/dL (DBil 1.7/IBil 0.5)	TBIL 0.2-1.2 mg/dL
Lt-CD4+	54 cells/mm³	493-1666 cells/mm³
HIV Viral Load	83 copies/mL	Not detected

Oral treatment was initiated with rifampin, isoniazid, pyrazinamide, and ethambutol (RHZE). On the third day of treatment, the patient evolved with elevated bilirubin levels (total bilirubin (TBil) of 3.3 mg/dL - direct bilirubin (DBil) of 2.8 mg/dL/indirect bilirubin (IBil) of 0.5 mg/dL) and was transferred to the intensive care unit (ICU). During the ICU period, the patient had multiple episodes of upper GI bleeding, requiring several red cell transfusions until stabilization. During this period, an intravenous alternative tuberculostatic scheme was prescribed for two weeks instead of oral medication, leading to sustained clinical improvement, with other episodes of GI bleeding.

**Figure 3:**
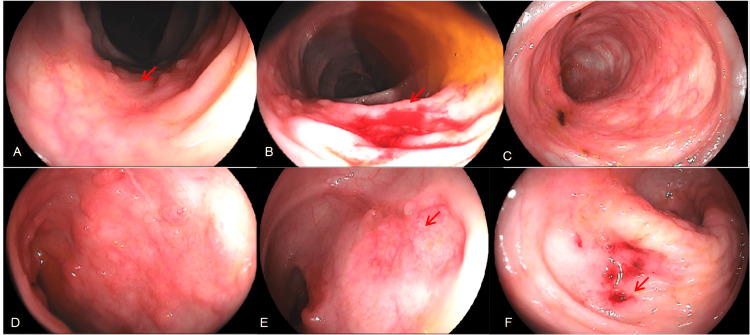
Upper endoscopy and colonoscopy A. An ulcerated lesion with a depressed center, a clean base, and irregular, granular, and nodular-appearing margins, measuring 2.0 cm in diameter, located in the transverse colon (red arrow). B. An ulcerated lesion with a depressed center and active bleeding, exhibiting slightly irregular, granular, and nodular margins, measuring 2.5 cm in diameter, located in the transverse colon (red arrow). C. An area with mucosal irregularity and retractions. D. An area of hyperemia with mucosal irregularity, cicatricial retractions, and granular and nodular scar-like appearance with a hematin spot present. E. An ulcerated lesion with a central depression, clean base, irregular margins, and a granular and nodular appearance, measuring 2.0 cm in diameter, located in the ascending colon (red arrow). F. A depressed ulcerated lesion with central bleeding foci, measuring 3.0 cm in diameter, located in the ascending colon (red arrow).

Upper endoscopy and colonoscopy were obtained to assess the source of the GI bleeding. The upper view revealed pangastritis with lesions on the gastric fundus, with active bleeding. Lower endoscopy showed multiple shallow ulcers with active pouring bleeding in the ascending and transverse portions of the colon (Figure 3).

**Figure 4:**
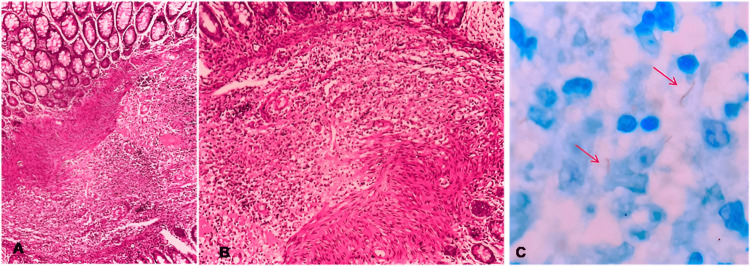
Staining for acid-fast bacilli A. Chronic inflammation demonstrated by histopathological findings in the lamina propria of the right colon (H and E 200X). B. Lymphohistiocytic aggregates, outlining granulomatous arrangements (H and E 400X). C. Presence of mycobacteria in the cytoplasm of macrophages (red arrows) (ZN 1000X). H and E: Hematoxylin eosin; ZN: Ziehl-Neelsen

Lesions were cauterized and biopsied. Anatomopathological analysis showed chronic colitis with outlined granulomas in the lamina propria, associated with positive Ziehl-Neelsen staining for acid-fast bacilli (Figure 4). Direct search for fungi was negative.

After sustained clinical improvement and control of bleeding, TB drugs were returned to the oral scheme, an oral diet was reintroduced, and the patient went to the infirmary for continuing care. Two weeks later, another episode of GI bleed presented with melena, with hemodynamic repercussions and the need for urgent transfusion protocol. The patient was once again sent to the ICU. This time, she evolved to refractory circulatory shock after multiple massive episodes of melena and, despite all efforts, died.

Case 2

**Figure 5:**
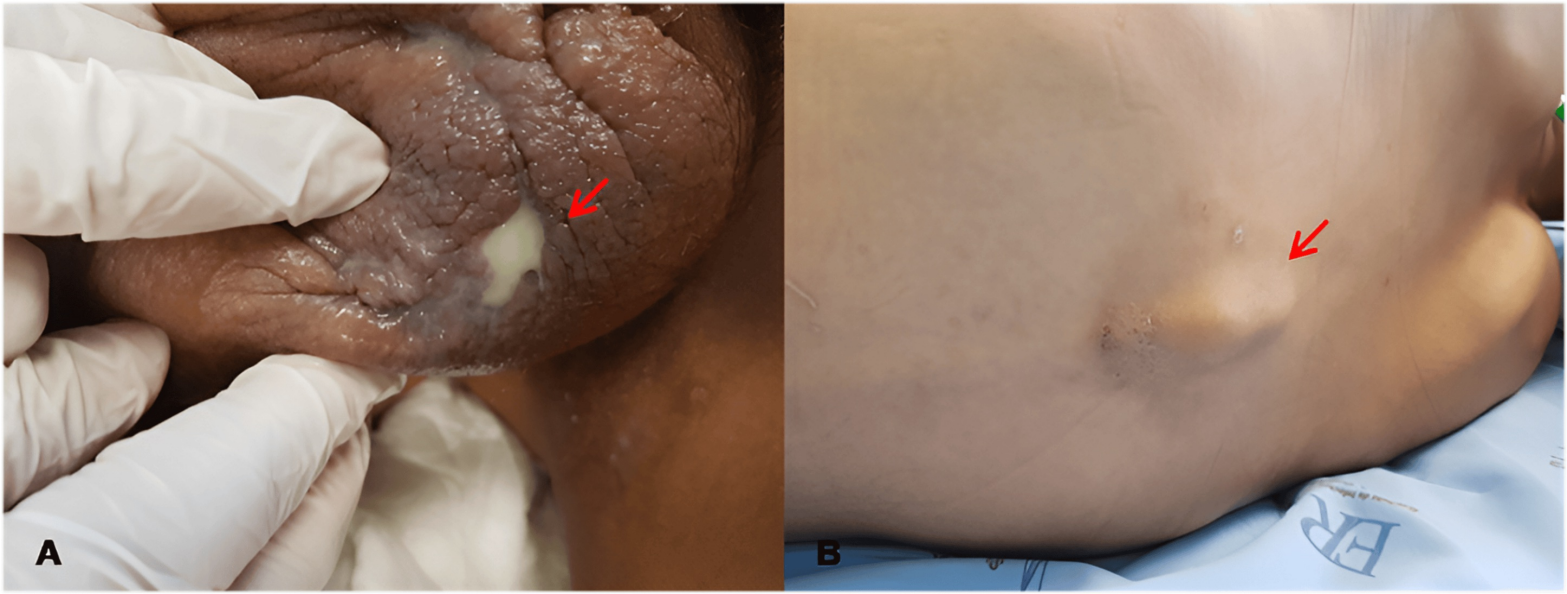
Case 2 - A. An inflammatory process in the scrotum, having cutaneous-scrotum fistulae with purulent secretion drainage with expression (red arrow). B. Patient’s back with a visible and palpable thoracic vertebral spine bulging (red arrow)

The second case we present, in contrast with Case 1, concerned a 24-year-old male with a history of untreated syphilis and tobacco use, with no known chronic illnesses. He went to the infectious disease emergency department with a three-month history of nonintentional 15 kg weight loss and three weeks of productive cough, night sweats, and low-grade fever. Concomitantly, he had pain, edema, and purulent secretion on his scrotum, which had evolved over the past three years (Figure 5A). Finally, he had developed, also in the last three years, a palpable growing nodule in his thoracic vertebral spine that had developed into a painful mass (Figure 5B).

The rapid HIV test yielded a negative result, while the results of the other laboratory tests conducted upon admission can be found in Table 2. The hemoglobin (8.4 g/dL) and hematocrit (26.8%) levels were significantly reduced, indicating anemia. C-reactive protein (CRP) was markedly elevated at 136 mg/L (reference: <50 mg/L), consistent with an active inflammatory process. All other parameters were within their respective reference ranges.

**Table 2:** Laboratory analysis on admission - Case 2 CRP: C-reactive protein; GPT: glutamate pyruvate transaminase; GOT: glutamic oxaloacetic transaminase; LDH: lactate dehydrogenase; WBC: white blood cells

Exam	Result	Reference Value
Hemoglobin	8.4 g/dL	13.5-17.5 g/dL
Hematocrit	26.8%	40.0-52.0%
WBC	8 x 10³/mm³	4.00-11.00 x 10³/mm³
Platelets	320 x 10³/mm³	140-450 10³/mm³
CRP	136 mg/L	< 50 mg/L
LDH	229 U/L	120-246 U/L
Creatinine	0.79 mg/dL	0.52-1.04 mg/dL
Urea	27 mg/dL	15-36 mg/dL
GOT	20 U/L	< 59 U/L
GPT	17 U/L	< 50 U/L

**Figure 6:**
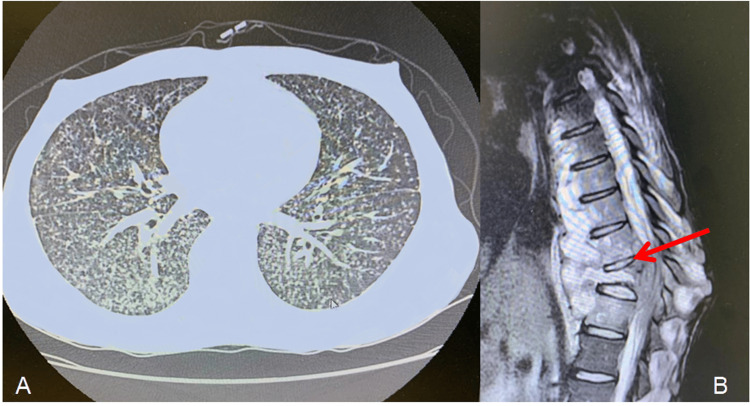
Patient's CT scan and MRI A. CT scan without intravenous contrast showing bilateral, diffuse, micronodular pulmonary involvement compatible with miliary TB. B. The MRI study showing the severe vertebral inflammatory process with vertebral collapse and paravertebral contrast enhancement (red arrow), compatible with Pott’s disease.

The chest CT revealed diffuse micronodular opacities in both lungs, compatible with miliary TB (Figure 6A), and sputum analysis was positive for acid-fast bacilli staining and scrotal purulent secretion. Both samples were submitted to molecular testing for TB and tested positive without rifampin-resistant gene detection. In addition, the CT study showed an upper thoracic process in the patient’s palpable back mass topography, compatible with an abscess. An advanced MRI study was compatible with Pott’s disease (Figure 6B). The patient was admitted, and oral therapy with RHZE for TB was started.

**Figure 7:**
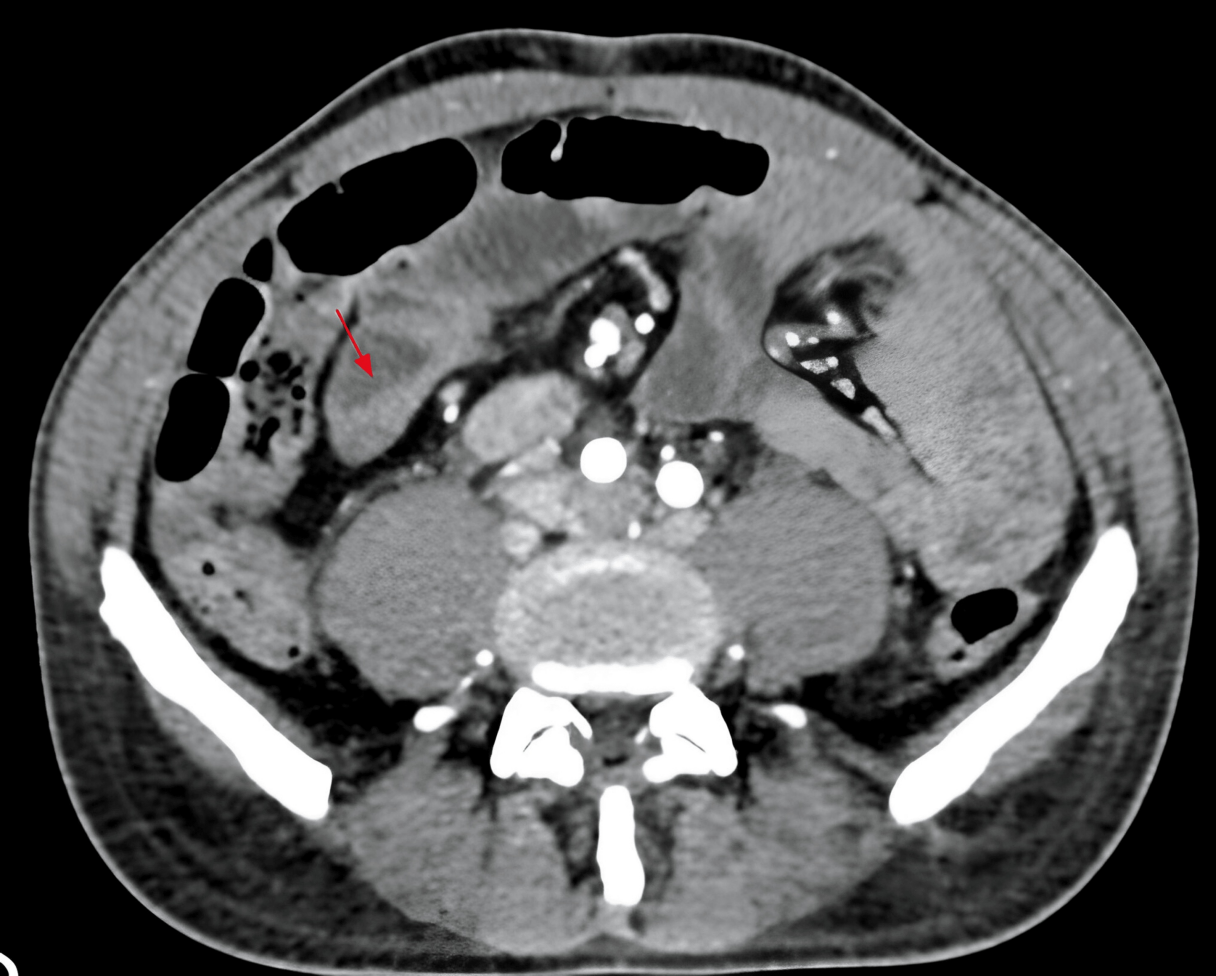
Abdominal tomography demonstrating ileal thickening (red arrow)

After six days of treatment, the patient presented with severe lower digestive bleeding, with hemoglobin levels reaching 2.9 mg/dL, circulatory shock, and hemodynamic instability, with an indication of ICU. An abdominal CT showed ileum thickening (Figure 7).

**Figure 8:**
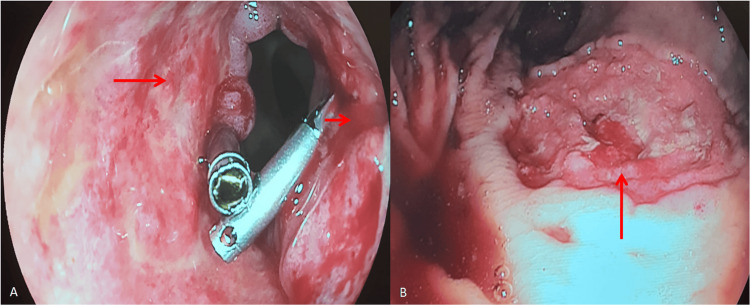
A. An ulcer affecting the distal ileum’s entire circumference, 5-cm long from the ileocecal valve, with active pouring bleeding (red arrows). B. Rectal ulcer 5 cm long, with active central bleeding (red arrow)

He needed multiple transfusions and vasoactive drugs. He reached clinical hemodynamic stability and was given a colonoscopy that identified two extensive ulcers, one surrounding the distal ileum wall and the other in the distal portion of the rectum, both with active bleeding and requiring local therapy. He had another episode, one week later, that showed a recurrence of the bleeding in the same spots as before (Figure 8).

With bleeding recurrence despite initial treatment, the patient was transitioned to full IV therapy, including IV TB treatment with levofloxacin, linezolid, amikacin, and meropenem, in addition to total parenteral diet due to suspicion of malabsorption and intestinal function impairment.

**Figure 9:**
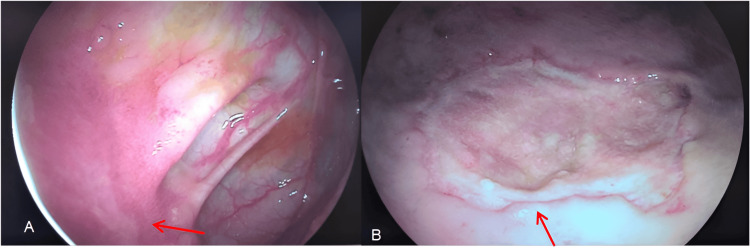
Colonoscopic control images after 15 days of IV therapy with evidence of fibrinous coverage and no bleeding in both ulcerated lesions A. An ulcer affecting the distal ileum (red arrow). B. A rectal ulcer (red arrow)

**Figure 10:**
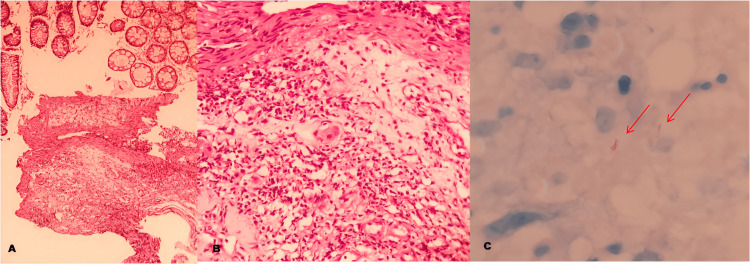
A. Lymphohistiocytic inflammatory infiltrate in the submucosa of the transverse colon (H and E 100x). B. Lymphohistiocytic inflammatory infiltrate in the submucosa of the transverse colon (H and E 400x). C. Presence of acid-fast bacilli indicated by red arrows (ZN 1000x) H and E: Hematoxylin eosin; ZN: Ziehl-Neelsen

After full IV transitioning, the patient did not have more episodes of GI bleed. A control colonoscopy was conducted 14 days after the last occurrence of bleeding and revealed both ulcers with fibrinous layers and no signs of recent bleeding (Figure 9). At that time, biopsies were conducted and later revealed acid-fast bacilli in staining (Figure 10). After one month, a novel colonoscopy was performed, showing that the ulcers were almost completely healed. The patient improved, and bleeding did not recur. He was discharged with outpatient follow-up after a total of three months of hospital care.

## Discussion

The most common complication of intestinal TB is obstruction. It occurs as a result of intra-abdominal adhesions that form in consequence of a chronic inflammatory and scarring process [6]. Spectant clinical treatment is the first course of action, surgery being reserved for those with an acute abdomen or refractory obstructions [4,13]. In some cases, nonetheless, despite high mortality rates, surgery may be the only viable treatment course [9].

GI hemorrhage secondary to intestinal TB is rare, although it represents a challenge given that there are no standardized protocols for its management [2,3].

Other cases reported had similar situations to our patients. The first was a 26-year-old patient with systemic erythematous lupus being treated with immunosuppressants, who was admitted to the ED with persistent GI bleed due to TB [13]. A parenteral diet was instituted with good evolution and outcome. The other case reported was a 28-year-old male renal transplant patient with hematochezia secondary to intestinal TB [12]. He received a right hemicolectomy with terminal ileostomy with good initial bleeding control, but had postoperative sepsis and died [12].

The cases presented above reflected different approaches to the same clinical situation and different outcomes. Because therapeutic strategies lack valid and well-established protocols, there is no standardized care plan for these patients with regard to choosing the course of nutrition and drug delivery or when to repeat control endoscopic exams. More studies need to assess this problem, and with enough data, a protocol should be designed focusing on the reduction of morbid and unsuccessful procedures and the adoption of those that can reduce the morbidity and mortality of this disease’s complications.

Our cases show differences in the recurrence of bleeding if exclusive IV drug and nutrition are prescribed compared with a recurrence after transition to oral therapy and clinical improvement without recurrence when antituberculous IV drugs were used.

The initial findings of a pouring bleed and the confirmation of TB, excluding other etiologies, raised concerns about the prognostic value of these patients’ clinical features [12,13].

In our first patient, an urgent colonoscopy exam at the beginning of the bleeding exteriorization could have led to a faster assessment of the bleeding source and its control [4]. After treatment and endoscopic therapy, a novel control exam could and should have been scheduled within a predetermined time frame, before the oral switch. Some studies show that endoscopic therapy at an opportune time leads to better outcomes in these situations, justifying the need for a proper care protocol [14].

Finally, the patient’s oral transition to anti-TB treatment could have contributed to the recurrence of her GI bleeding and death. Malabsorption, frequently related to TB’s intestinal impairment, as well as other inflammatory intestinal diseases, could have limited the oral drug absorption and, thus, its biodisponibility, resulting in lesion persistence and rebleeding. In contrast, the second case remained on IV therapy until a follow-up endoscopic examination confirmed complete healing of the ileal and rectal ulcers. Only thereafter was oral therapy reintroduced, with the patient subsequently showing clinical improvement and being discharged for outpatient follow-up. Studies show that the use of IV tuberculostatic drugs has been successful in cases of intestinal TB, especially in cases of high disease burden, circulatory shock, and absorption impairment [10,11].

## Conclusions

These two cases, although highlighting a common co-infection (HIV+ disseminated TB), also underscore a rare but severe and potentially fatal complication of intestinal TB. Episodes of massive GI bleeding, leading to death in both immunocompromised and immunocompetent patients, reinforce the importance of early diagnosis and active surveillance across these populations. Individualized endoscopic treatments and the initial use of the IV route, reserving oral therapy for a later stage, are simple yet potentially effective measures that may improve patient outcomes. However, there is currently no strong evidence in the literature with statistical analysis supporting these strategies, emphasizing the need for further studies on this subject.
